# Over-the-scope clip for closure of persistent gastrocutaneous fistula after gastrostomy tube removal: a multicenter pediatric experience

**DOI:** 10.1007/s00464-024-11166-2

**Published:** 2024-08-26

**Authors:** Antonio Corsello, Matthieu Antoine, Shishu Sharma, Valérie Bertrand, Salvatore Oliva, Giorgio Fava, Francesca Destro, Andrew Huang, Wei S. W. Fong, Martina Ichino, Mike Thomson, Frederic Gottrand

**Affiliations:** 1 Division of Gastroenterology, Hepatology and Nutrition, Department of Paediatrics, University of Lille, Inserm, CHU Lille, U1286 - INFINITE, Lille, France; 2 University of Milan, Milan, Italy; 3 Centre for Paediatric Gastroenterology, Sheffield Children’s Hospital NHS Foundation Trust, Sheffield, UK; 4 Pediatric Unit, Le Havre Hospital, Le Havre Cedex, France; 5 Pediatric Gastroenterology and Liver Unit, Department of Maternal and Child Health, Sapienza University of Rome, Rome, Italy; 6 Department of Pediatric Surgery, Fondazione IRCCS Ca’ Granda Ospedale Maggiore Policlinico, Milan, Italy; 7 Department of Pediatric Surgery, Buzzi Children’s Hospital, Milan, Italy; 8 Division of Pediatric Gastroenterology, Hepatology and Nutrition, Department of Pediatrics, University of Nebraska Medical Center and Children’s Hospital & Medical Center, Omaha, NE USA; 9 Service d’hépato, gastroentérologie et nutrition pédiatrique, Pôle enfant, Hôpital Jeanne de Flandre, Avenue Eugène Avinée, Lille, France

**Keywords:** OTSC, Ovesco, Gastrocutaneous fistula, PEG removal, Pediatric surgery, Closure techniques

## Abstract

**Background:**

Percutaneous endoscopic gastrostomy is commonly used for enteral nutritional access, but gastrocutaneous fistulae (GCF) may persist after tube removal, posing clinical challenges. The use of endoscopic closure devices, including over-the-scope clips (OTSC), has shown promise in managing non-healing fistulae, although data in the pediatric population are limited.

**Methods:**

A retrospective multicenter study analyzed pediatric patients who underwent GCF closure following gastrostomy tube removal. Data from seven centers across multiple countries were collected, including patient demographics, procedural details, complications, and outcomes. Closure techniques were compared between OTSC and surgical closure.

**Results:**

Of 67 pediatric patients included, 21 underwent OTSC closure and 46 had surgical closure. Surgical closure demonstrated a higher success rate (100%) compared to OTSC closure (61.9%, *P* < 0.001). While procedural duration was shorter for OTSC closure (25 vs. 40 min, *P* = 0.002), complications, and scar quality were comparable between techniques. A subsequent sub-analysis did not reveal differences based on center experience.

**Conclusion:**

OTSC closure is feasible and safe in pediatric patients, but surgical closure remains superior in achieving sustained GCF closure, although OTSC offers benefits, such as shorter procedural duration, potentially reducing the duration of general anesthesia exposure. Non-operative approaches, including OTSC, may be a valuable alternative to surgical closure.

**Graphical abstract:**

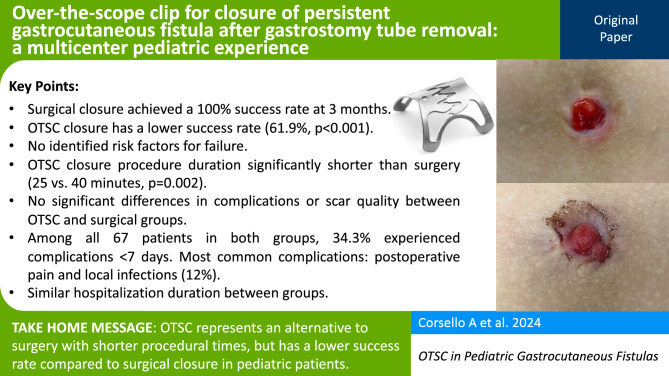

Percutaneous endoscopic gastrostomy (PEG) has become a common procedure for establishing enteral nutritional access in patients unable to maintain adequate oral intake, even if modern laparoscopic techniques represent valid alternatives [1–3]. Both approaches can serve as a temporary or permanent solution and offering an alternative route for nutritional support [3, 4]. Resuming normal oral nutrition, especially in children, often allows the gastrostomy to be removed. The standard removal of tubes is typically straightforward and can be performed in a clinical outpatient setting and spontaneous closure of the gastrostomy tract may take up to 1–2 weeks. However, a gastrocutaneous fistula (GCF) may persist even for months, posing clinical complications, such as local infections or major leaks [5–7]. The occurrence of persistent (> 1–3 months) GCF ranges from 4.5% to 44%, depending on various risk factors and definitions [6, 8–11]. Age at gastrostomy placement and timing of removal are recognized as predisposing factors for GCF, as well as prolonged non-use or extended tube retention time. Indeed, one-third of children with predictable risk factors including age at gastrostomy and length of time of tube retention face challenges in managing the tube removal, mainly due to persistent GCF [9]. Moreover, cauterization and outpatient procedures may be frequently unsuccessful in persistent GCF management, and surgical closure is then often required [10].

The emergence of endoscopic closure devices in recent years has revolutionized the approach to non-healing fistulae, marking a shift away from traditional surgical interventions. Novel techniques, including endoscopic clips, percutaneous sutures, skin glues, and balloon catheters, have shown promise in reducing complications, shortening hospital stays, and facilitating the resumption of oral feeding [12, 13]. Experience in non-operative management of persistent GCF in children remains scarce; a systematic review of 142 cases reported an 80% success rate, with endoscopy showing a 75% success rate, with no specific adverse events [9].

Among the spectrum of endoscopic closure devices, over-the-scope clips (OTSC) have gained prominence as a versatile tool in several indications, including persistent GCF (Fig. 1) [14–18]. Originally developed for adult endoscopic practice, the OTSC has demonstrated long-term efficacy of > 50% in a large range of applications, from acute hemostasis to various kinds of fistulae of the whole gut [12, 15, 19, 20]. Even so, the application of OTSC in the pediatric population, especially in the context of post-gastrostomy fistulae, remains poorly reported [21].

**Fig. 1:**
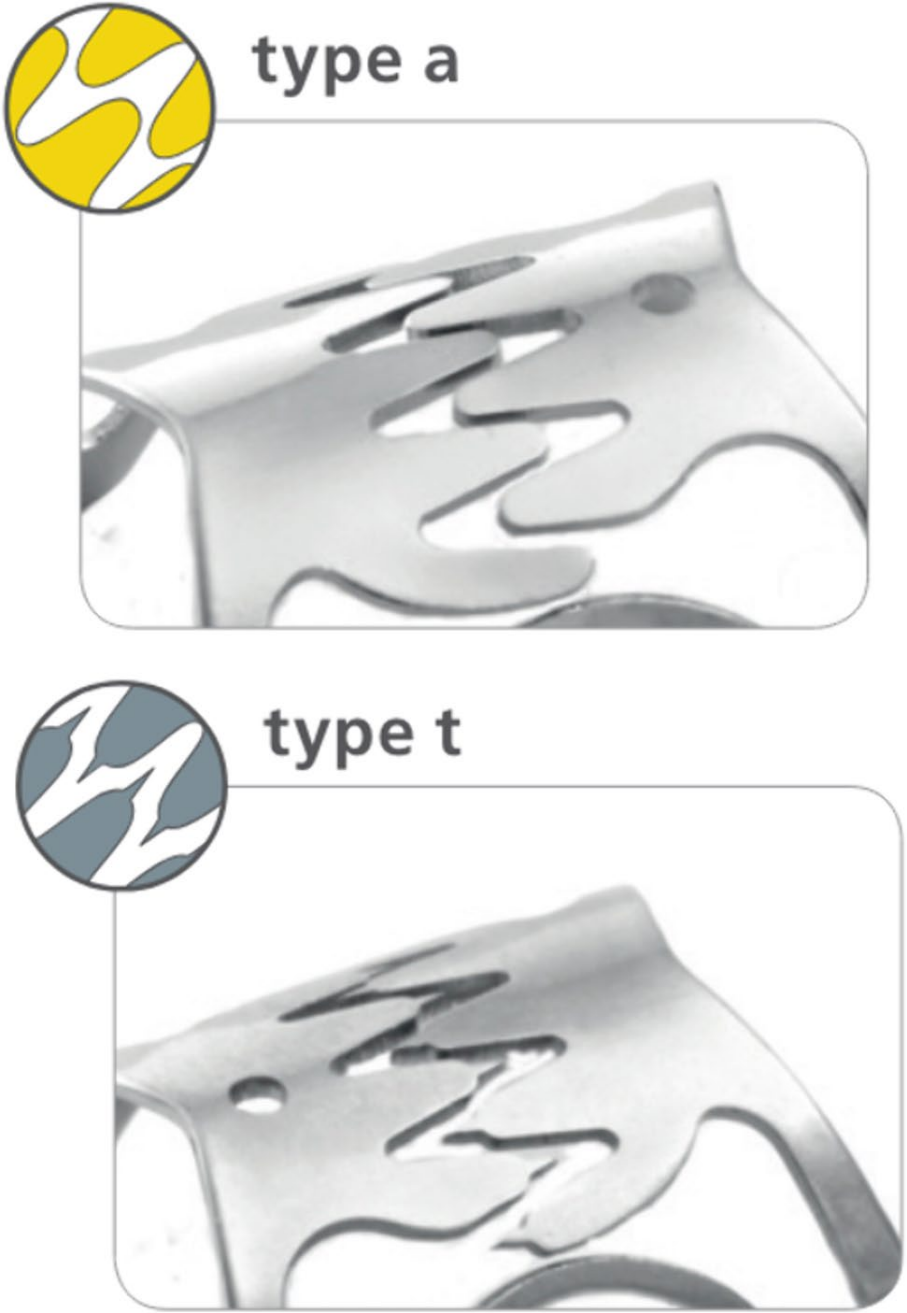
Most used over-the-scope clip (OTSC) devices and their specifics [36]. The “a clip” with round teeth is used if blunt compression of the tissue is intended. The “t clip” has spikes and is used if additional anchoring of the clip is intended, e.g., in fibrotic tissue. A standard 9–10-mm pediatric gastroscope was typically used for the OTSC procedure, accommodating the deployment of 11-mm or 12-mm clips

Given the limited pediatric data available on the application of OTSC in post-gastrostomy fistulae, our study aimed to investigate the management and outcomes of GCF procedural closure following the removal of the tube and to compare the outcomes of OTSC closure (Figs. 2 and 3) with those of traditional surgical closure.

**Fig. 2:**
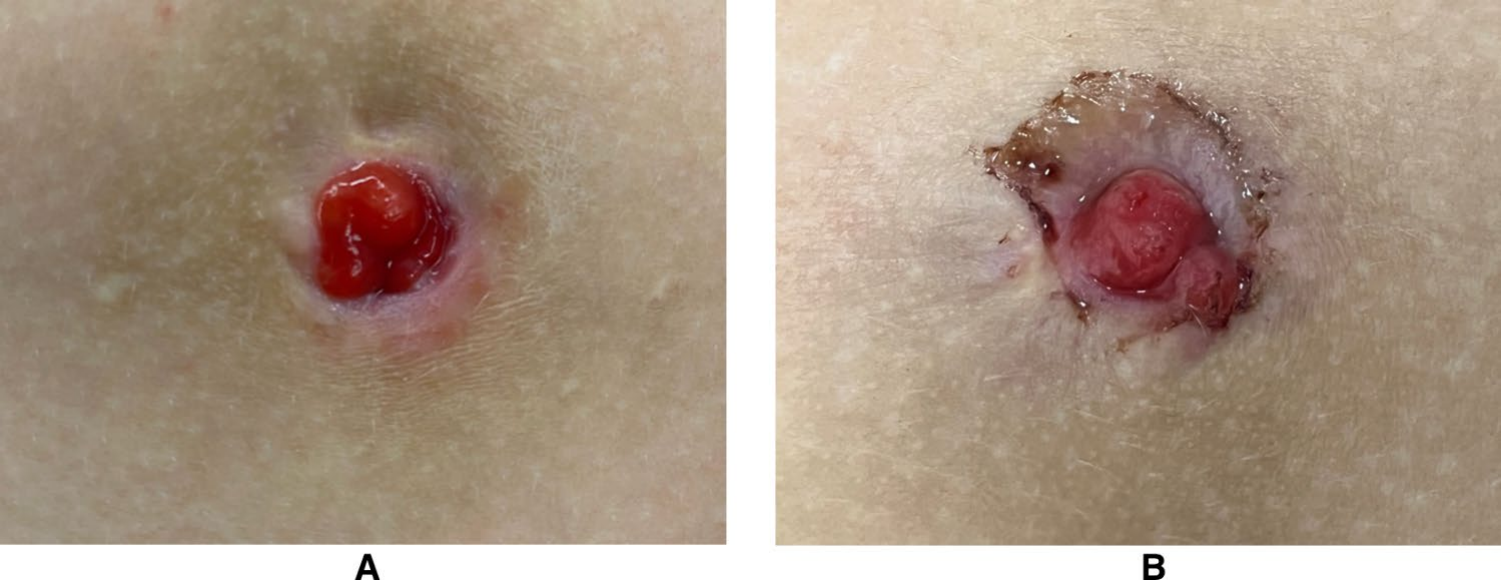
Persistent gastrocutaneous fistula at the time of the closure with over-the-scope clip (OTSC) (**A**) and at 8 months later (**B**), when biopsies of the mucous bud found gastric mucosa

**Fig. 3:**
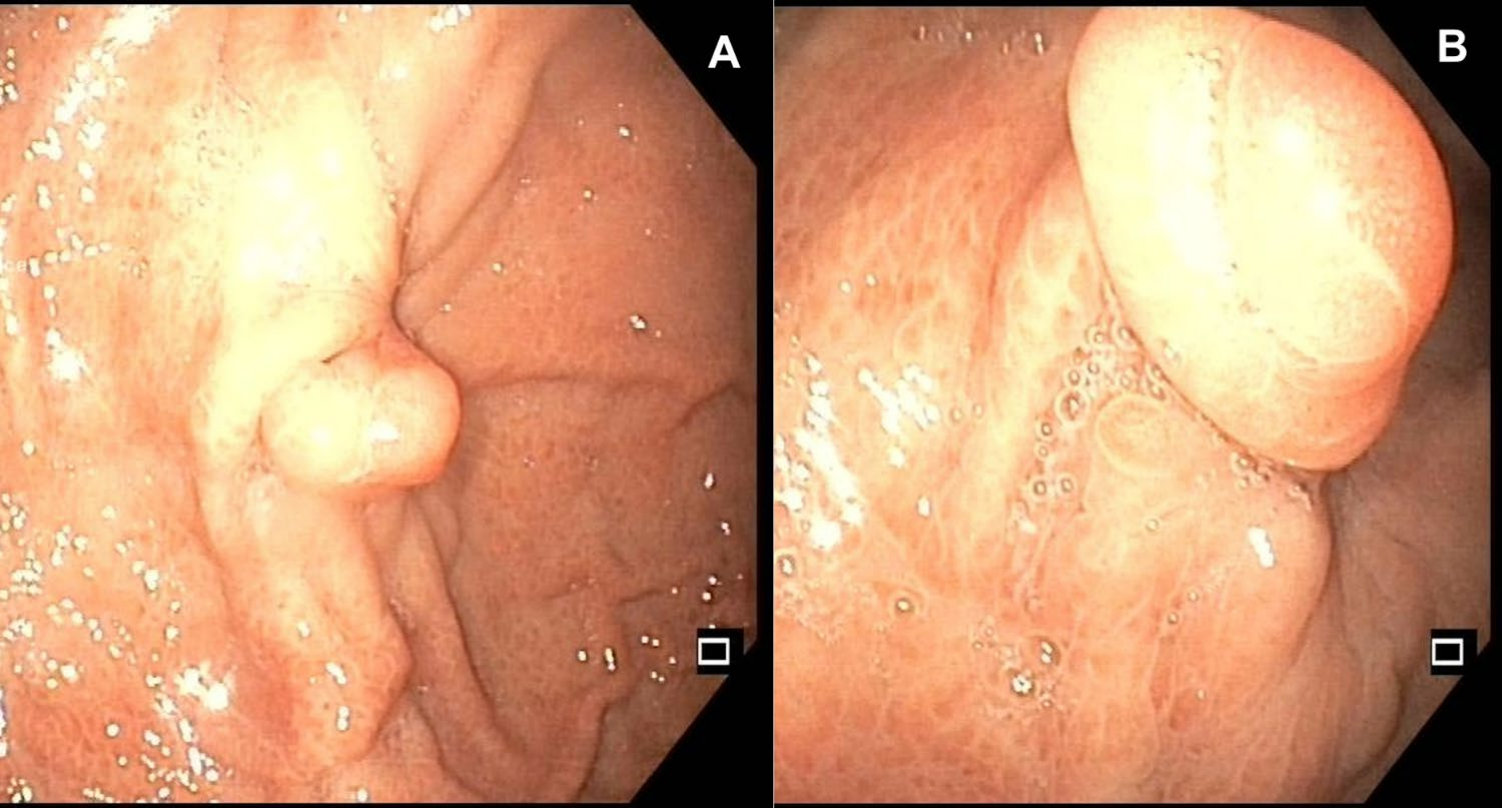
Endoscopic aspect of an over-the-scope clip (OTSC) closure (**A**), 8 months after the procedure (**B**)

## Materials and methods

This retrospective multicenter study included pediatric patients who underwent closure of GCF following tube removal from June 2014 to June 2023. Data were collected from seven centers: Lille (France), Sheffield (United Kingdom), Le Havre (France), Milan Ospedale Maggiore Policlinico (Italy), Milan Buzzi Children Hospital (Italy), Rome (Italy), and Omaha (Nebraska, USA).

We included all pediatric patients (< 18 years) who underwent closure for GCF, with a minimum follow-up of 3 months. Clinical data were retrospectively extracted from medical records in an anonymized electronic case report form. The collected variables included patient demographics (sex, age at removal, age at fistula closure), procedural details (time intervals between gastrostomy placement, removal, and fistula closure), reasons for gastrostomy insertion, and underlying conditions (e.g., neuromuscular diseases, severe food aversion). The type of gastrostomy was determined, including Push One-Step PEG, Pull-Through PEG, or others (e.g., surgical or radiologic placement).

Specific information about the OTSC procedure was recorded, including the type, size, and depth of the OTSC, as well as any technical difficulties. Additional procedural aspects, such as the need for grasper or anchor forceps, the use of extra devices, and the time required for OTSC placement, were documented. Early outcomes were assessed, including days of hospitalization after the procedure, weight at fistula closure, and associated drugs. Complications were categorized as immediate (during the procedure), early (within 7 days), and late (7 days or after). Complications including pain, bleeding, local or systemic infections, and modalities for the management of complications were recorded. The success or failure of the closure was documented, with success defined as closure of the fistula confirmed 3-month post-procedure. Patients from the same centers of comparable age, who presented persistent GCF and underwent surgical closure, were chosen as a control group. The scar quality was assessed by querying caregivers at least 3-month post-procedure regarding their perception of its esthetic appearance (good, fair, poor).

The study was conducted using protocols, good clinical practice, and relevant laws and regulations. This study was approved by the Ethics Committee of the Gastroenterology, Hepatology, and Nutrition French Speaking Group (ID: 2023-45). The study was declared to the Data Protection Authority and conducted according to the Helsinki Declaration. All data were anonymized.

Study data were collected and managed using REDCap® electronic data capture tools hosted at “Centre Hospitalier Universitaire de Lille” in France. Descriptive statistics were presented as mean (standard deviation) or median (interquartile range (IQR)) in the case of non-normal distribution for quantitative variables and as frequency and percentage for categorical variables. The normality of distribution was assessed graphically and using the Shapiro–Wilk test. Bivariate comparison between the OTSC and surgical closure groups was performed using the Chi-square test or Fisher’s exact test for categorical variables and the Mann–Whitney *U* test for quantitative variables. Statistical testing was conducted at the two-tailed *α* level of 0.05. Data were analyzed using SAS software (version 9.4; SAS Institute, Cary, NC).

## Results

### Patients’ characteristics

In total, 67 pediatric patients with GCF were included. Twenty-one patients underwent OTSC placement and 46 with a surgical closure (Table 1).

**Table 1:** Comparison of the population according to the type of GCF closure

		Fistula closure, *N* (%)		
		OTSC *N* = 21	Surgery *N* = 46	*P value*
Characteristics				
Sex	Male	11 (52.4)	18 (39.1)	0.31
	Female	10 (47.6)	28 (60.9)	
Type of gastrostomy insertion	Push	7 (33.3)	17 (37.0)	
	Pull	7 (33.3)	11 (23.9)	
	Other	7 (33.3)	18 (39.1)	
Gastrostomy indication	Inadequate nutritional intake	18 (85.7)	39 (84.8)	1.00
	Unsafe swallow	9 (42.9)	13 (28.3)	0.24
	Other	4 (19.0)	14 (30.4)	0.33
Underlying disease	Food aversion/eating disorders	5 (23.8)	12 (26.1)	0.84
	Neurological impairment	12 (57.1)	18 (39.1)	0.17
Tube removal indication	Gastrostomy no longer needed	16 (76.2)	37 (80.4)	0.75
	Major leaks at the gastrostomy	7 (33.3)	14 (30.4)	0.81
	Other	5 (23.8)	14 (30.4)	0.58
Duration of gastrostomy in place	Months (IQR)	62 (35.9–139.2)	51.2 (30.5–78.3)	0.24
Time between removal and fistula closure	Months (IQR)	2.8 (0.8–5.8)	4.7 (2.1–8.8)	0.13
Age at fistula closure	Years (IQR)	8 (5.4–16.7)	6.5 (4–9.9)	0.14
GCF closure procedure				
Outcome	Success	13 (61.9)	46 (100.0)	< 0.001
Closure procedure duration	Min (IQR)	25 (18–30)	40 (30–48)	0.002
Hospitalization duration	Days (IQR)	1 (1–2)	1 (1–2)	0.32
Post-procedure complications	Yes	6 (28.6)	17 (37.0)	0.50
Need for complication management	Yes	7 (33.3)	7 (15.2)	0.11
Quality of the scar	Good	7 (58.3)	17 (70.8)	
	Fair	3 (25.0)	6 (25.0)	
	Bad	2 (16.7)	1 (4.2)	

*GCF* gastrocutaneous fistula, *OTSC* over-the-scope clips, *IQR* interquartile range

The median age at fistula closure for the entire cohort was 7.1 years (IQR 4.5–11.5), with a median time of 4.3 months (IQR 1.4–8.2) between tube removal and fistula closure. Considering the 67 patients, 62.7% had their gastrostomy placed via PEG, while 32.8% had it placed surgically (open/laparoscopic). Overall, patients maintained the gastrostomy for an average of 4.9 years (IQR 2.7–7). No significant statistical difference was observed between the two groups regarding the median time from the removal to the fistula closure (2.8 vs. 4.7 months, *P* = 0.13) and gastrostomy retention time (62 vs. 51.2 months, *P* = 0.24). In addition, no significant differences were detected across factors, such as procedural challenges, patient sex, underlying conditions, age, or weight at the time of closure.

### OTSC closure: technical aspects

The majority (66.7%) of OTSC applied were 11 mm in diameter, while the remaining clips were 12 mm. In most cases (61.9%), the clip depth was 6 mm. Type t clips were preferred rather than type a (85.7% vs. 14.3%). In patients who underwent fistula closure with OTSC, an average procedure duration of 25 min was observed, and no technical difficulties or complications were encountered during clip application.

### Comparison of outcome of OTSC and surgical closure

Surgical closure was successful in 100% of cases at 3 months of follow-up, whereas OTSC closure demonstrated a 61.9% success rate (*P* < 0.001). Indeed, we observed 8 cases of closure failure with OTSC postoperatively (*n* = 3), within 1 week (*n* = 3), and within one month after the procedure (*n* = 2). We could not find any risk factor associated with failure in the OTSC group.

The duration of the closure procedure was significantly shorter in the OTSC group than in the surgery group (25 min vs. 40 min, *P* = 0.002) (Table 1).

When considering the occurrence of complications and scar quality, no significant differences were found between the two groups (Fig. 2). Considering all 67 patients, 23 (34.3%) experienced complications. No intra-procedural or late (≥ 7 days) complications were found. The most common early complications were postoperative pain and local wound infections, occurring in 12% of cases. No significant difference was found in hospitalization duration between the two groups.

To address a possible bias related to the variable experience of the centers and learning curves effect on OTSC placement, a sub-analysis was conducted comparing 12 OTSCs performed in the most experienced centers (> 3 OTSC closures already performed) with the 9 cases from less-experienced centers, or early cases from experienced centers. We could not find any difference either in success or in complication rates (*P* = 0.67).

## Discussion

Our results show that OTSC is feasible and safe in children as young as 2 years or weighing more than 10 kg. To the best of our knowledge, our study is the first to compare OTSC with surgical closures of GCF in children and shows that surgery is superior in achieving sustained closure of GCF, with a persistent fistula occurring in more than 1/3 of the OTSC closure group. The shorter procedural duration for OTSC compared with surgery aligns with the existing literature on the potential advantages of GI endoscopic techniques in terms of procedural efficiency and duration, which our results confirm [22, 23]. Indeed, while endoscopic closure is a viable technique, its selection should be based on individual patient circumstances. Our findings suggest that surgical closure should remain the standard of care for persistent GCF in pediatric age, with OTSC serving as an alternative when surgery is contraindicated or has failed.

Nonetheless, it is useful to consider the benefits of OTSC beyond just numerical outcomes, especially its role as a minimally invasive approach in addressing GCF [24]. Potentially, OTSC and other non-operative methods could offer significant advantages over traditional surgical options, including reduced complication rates, shorter hospital stays, and faster return to oral feeding [9, 25–27]. However, it is important to clarify that while OTSC is less invasive than surgery, it still requires general anesthesia in pediatric patients. This necessity ensures the safety and immobility of the pediatric patient during the procedure, similar to the surgical approach [21, 28]. Thus, the primary advantage of OTSC could be related to its reduced procedural duration and a potentially quicker postoperative recovery, rather than the avoidance of general anesthesia. Moreover, given the growing concern over the neurodevelopmental effects associated with general anesthesia in pediatric patients, the ability to minimize procedural time could be an advantage [29, 30]. However, recent evidence suggests no increased risk associated with general anesthesia for procedures under 1 h, even in younger pediatric patients [31]. Consequently, a 15-min difference in procedural time between OTSC and surgical closure might not be clinically significant in terms of anesthesia exposure risk, particularly when considering the higher success rate associated with surgical closure.

The shorter duration of the endoscopic procedure confirmed by our results emphasizes the need for prompt effectiveness and suitable safety of non-operative techniques. A systematic review suggests that such non-operative modalities have shown promise [9], especially in avoiding unnecessary or longer exposure to general anesthesia in vulnerable pediatric patients [32–34]. There are limited pediatric data on the application of OTSC while existing evidence primarily pertains to adults. Our study is the largest series of OTSC applications in children (*n* = 21) and confirms the applicability and functional results of previous reports. Sharma et al. reported the clinical success of OTSC closure in site fistulae in 6 of 7 children [21]. Wright et al. reported clinical success in 5 of 6 children [18]. In terms of complications, scar quality, and hospitalization duration, both techniques offer comparable safety profiles. Moreover, in cases of a first-line endoscopic approach, OTSC would not interfere with an eventual future surgery, if needed [35].

While OTSC offers advantages, such as reduced procedural duration and potentially decreased hospital stay, it should be viewed as an option rather than the first treatment for pediatric GCF. Thus, the use of OTSC in pediatric patients should be considered on a case-by-case basis, particularly when standard approaches have failed to close the fistula or when specific clinical conditions make conventional techniques less suitable. This approach provides a balanced perspective on integrating OTSC into pediatric care, recognizing the need to tailor interventions to individuals.

The strength of our study lies in its multicenter design, collecting data from seven different centers. This approach enhances the generalizability of our findings, as it considers diverse patient populations, settings, and varying clinical practices. Indeed, the lack of significant differences in success rates or complications between more- and less-experienced centers in the OTSC cases underscores the consistency and reliability of this new endoscopic device across diverse settings, both in terms of case history and various surgical/pediatric competencies. Inherent limitations of this study include the potential for selection bias in treatment modalities and reliance on retrospective data with varying levels of GCF severity and underlying diseases. Although a 3-month follow-up was selected to define the success or failure of the GCF closure, we acknowledge that a longer follow-up period could capture additional late recurrences. However, extending follow-up might increase the likelihood of losing patients, thus limiting the reliability of long-term data. Therefore, while our current data provide valuable insights, further studies with extended follow-up would be useful to rule out the risk of late recurrence of the fistula. We acknowledge that variability in the techniques used across our patient cohort and among different centers could influence the results. This heterogeneity presents a limitation in our statistical analysis, potentially affecting the robustness of the comparisons between the OTSC and surgical closure groups. Another limitation of our study is that we could not identify any at-risk groups of children where OTSC was likely to fail, although it is possible to hypothesize that certain characteristics of the gastrostomy fistula (diameter, colonization with gastric mucosa, as shown in Figs. 2 and 3) might influence the outcome.

Even if OTSC closure demonstrates feasibility and safety in pediatric patients, surgical closure maintains superiority in achieving sustained closure of GCF in pediatric patients, with a 100% success rate in our cohort. Our study prompts further considerations for the integration of endoscopic techniques such as OTSC into the pediatric clinical practice, always balancing the benefits and risks of each approach for the specific patient and considering surgery in cases of stable and suitable patients.

## References

[1] Kvello M, Knatten CK, Bjørnland K (2020) Laparoscopic gastrostomy placement in children has few major, but many minor early complications. Eur J Pediatr Surg 30:548–553. 10.1055/s-0039-3401988 31891947 10.1055/s-0039-3401988

[2] Suksamanapun N, Mauritz FA, Franken J, van der Zee DC, van Herwaarden-Lindeboom MY (2017) Laparoscopic versus percutaneous endoscopic gastrostomy placement in children: results of a systematic review and meta-analysis. J Minim Access Surg 13:81–88. 10.4103/0972-9941.181776 27251841 10.4103/0972-9941.181776 PMC5363129

[3] Homan M, Hauser B, Romano C, Tzivinikos C, Torroni F, Gottrand F, Hojsak I, Dall’Oglio L, Thomson M, Bontems P, Narula P, Furlano R, Oliva S, Amil-Dias J (2021) Percutaneous endoscopic gastrostomy in children: an update to the ESPGHAN position paper. J Pediatr Gastroenterol Nutr 73:415–426. 10.1097/MPG.0000000000003207 34155150 10.1097/MPG.0000000000003207

[4] Romano C, van Wynckel M, Hulst J, Broekaert I, Bronsky J, Dall’Oglio L, Mis NF, Hojsak I, Orel R, Papadopoulou A, Schaeppi M, Thapar N, Wilschanski M, Sullivan P, Gottrand F (2017) European society for paediatric gastroenterology, hepatology and nutrition guidelines for the evaluation and treatment of gastrointestinal and nutritional complications in children with neurological impairment. J Pediatr Gastroenterol Nutr 65:242–264. 10.1097/MPG.0000000000001646 28737572 10.1097/MPG.0000000000001646

[5] El-Rifai N, Michaud L, Mention K, Guimber D, Caldari D, Turck D, Gottrand F (2004) Persistence of gastrocutaneous fistula after removal of gastrostomy tubes in children: prevalence and associated factors. Endoscopy 36:700–704. 10.1055/s-2004-825662 15280975 10.1055/s-2004-825662

[6] Lalanne A, Gottrand F, Salleron J, Puybasset-Jonquez AL, Guimber D, Turck D, Michaud L (2014) Long-term outcome of children receiving percutaneous endoscopic gastrostomy feeding. J Pediatr Gastroenterol Nutr 59:172–176. 10.1097/MPG.0000000000000393 24709828 10.1097/MPG.0000000000000393

[7] Bratu I, Bharmal A (2011) Incidence and predictors of gastrocutaneous fistula in the pediatric patient. ISRN Gastroenterol 2011:686803. 10.5402/2011/686803 21991525 10.5402/2011/686803 PMC3168482

[8] Stephenson KJ, Bonasso PC, Vasquez IL, Burford JM, Wyrick DL, Bhavaraju A, Dassinger MS (2022) An evaluation of pediatric gastrocutaneous fistula closure through the punch excision of epithelized tract procedure. Am Surg 88:1822–1826. 10.1177/00031348221084945 35420922 10.1177/00031348221084945

[9] St-Louis E, Safa N, Guadagno E, Baird R (2018) Gastrocutaneous fistulae in children—a systematic review and meta-analysis of epidemiology and treatment options. J Pediatr Surg 53:946–958. 10.1016/j.jpedsurg.2018.02.022 29506816 10.1016/j.jpedsurg.2018.02.022

[10] Janik TA, Hendrickson RJ, Janik JS, Landholm AE (2004) Analysis of factors affecting the spontaneous closure of a gastrocutaneous fistula. J Pediatr Surg 39:1197–1199. 10.1016/j.jpedsurg.2004.04.007 15300526 10.1016/j.jpedsurg.2004.04.007

[11] Gordon JM, Langer JC (1999) Gastrocutaneous fistula in children after removal of gastrostomy tube: incidence and predictive factors. J Pediatr Surg 34:1345–1346. 10.1016/S0022-3468(99)90008-8 10507426 10.1016/s0022-3468(99)90008-8

[12] Kobara H, Mori H, Nishiyama N, Fujihara S, Okano K, Suzuki Y, Masaki T (2019) Over-the-scope clip system: a review of 1517 cases over 9 years. J Gastroenterol Hepatol 34:22–30. 10.1111/jgh.14402 30069935 10.1111/jgh.14402

[13] Mennigen R, Senninger N, Laukoetter MG (2014) Novel treatment options for perforations of the upper gastrointestinal tract: endoscopic vacuum therapy and over-the-scope clips. World J Gastroenterol 20:7767–7776. 10.3748/wjg.v20.i24.7767 24976714 10.3748/wjg.v20.i24.7767 PMC4069305

[14] Masaki S, Yamada K (2021) Over-the-scope clip closure of persistent gastrocutaneous fistula after percutaneous endoscopic gastrostomy tube removal: a report of two cases. Cureus 13:e13206. 10.7759/cureus.13206 33728166 10.7759/cureus.13206 PMC7946610

[15] Singhal S, Changela K, Culliford A, Duddempudi S, Krishnaiah M, Anand S (2015) Endoscopic closure of persistent gastrocutaneous fistulae, after percutaneous endoscopic gastrostomy (PEG) tube placement, using the over-the-scope-clip system. Therap Adv Gastroenterol 8:182–188. 10.1177/1756283X15578603 26136836 10.1177/1756283X15578603 PMC4480569

[16] Heinrich H, Gubler C, Valli PV (2017) Over-the-scope-clip closure of long lasting gastrocutaneous fistula after percutaneous endoscopic gastrostomy tube removal in immunocompromised patients: a single center case series. World J Gastrointest Endosc 9:85–90. 10.4253/wjge.v9.i2.85 28250901 10.4253/wjge.v9.i2.85 PMC5311477

[17] Bartell N, Bittner K, Kaul V, Kothari TH, Kothari S (2020) Clinical efficacy of the over-the-scope clip device: a systematic review. World J Gastroenterol 26:3495–3516. 10.3748/wjg.v26.i24.3495 32655272 10.3748/wjg.v26.i24.3495 PMC7327783

[18] Wright R, Abrajano C, Koppolu R, Stevens M, Nyznyk S, Chao S, Bruzoni M, Wall J (2015) Initial results of endoscopic gastrocutaneous fistula closure in children using an over-the-scope clip. J Laparoendosc Adv Surg Tech A 25:69–72. 10.1089/lap.2014.0379 25531644 10.1089/lap.2014.0379 PMC4361278

[19] Kirschniak A, Kratt T, Stüker D, Braun A, Schurr M-O, Königsrainer A (2007) A new endoscopic over-the-scope clip system for treatment of lesions and bleeding in the GI tract: first clinical experiences. Gastrointest Endosc 66:162–167. 10.1016/j.gie.2007.01.034 17591492 10.1016/j.gie.2007.01.034

[20] Goenka MK, Rai VK, Goenka U, Tiwary IK (2017) Endoscopic management of gastrointestinal leaks and bleeding with the over-the-scope clip: a prospective study. Clin Endosc 50:58–63. 10.5946/ce.2016.028 27802375 10.5946/ce.2016.028 PMC5299974

[21] Sharma S, Barakat M, Urs A, Campbell D, Rao P, Schluckebier D, Gugig R, Thomson M (2022) Applicability, efficacy, and safety of over-the-scope clips in children. Gastrointest Endosc 95:489–499. 10.1016/j.gie.2021.10.011 34662583 10.1016/j.gie.2021.10.011

[22] Kumar N, Larsen MC, Thompson CC (2014) Endoscopic management of gastrointestinal fistulae. Gastroenterol Hepatol (N Y) 10:495–452 28845140 PMC5566192

[23] Albers DV, Kondo A, Bernardo WM, Sakai P, Moura RN, Silva GLR, Ide E, Tomishige T, de Moura EGH (2016) Endoscopic versus surgical approach in the treatment of Zenker’s diverticulum: systematic review and meta-analysis. Endosc Int Open 4:E678-686. 10.1055/s-0042-106203 27556078 10.1055/s-0042-106203 PMC4993875

[24] Bhurwal A, Mutneja H, Tawadross A, Pioppo L, Brahmbhatt B (2020) Gastrointestinal fistula endoscopic closure techniques. Ann Gastroenterol 33:554–562. 10.20524/aog.2020.0543 33162732 10.20524/aog.2020.0543 PMC7599355

[25] Kan SW, Huang T-Y, Ma H-P, Tay MZ, Tam K-W, Tsai T-Y (2022) Early versus delayed feeding after therapeutic endoscopic procedures: meta-analysis of randomized controlled trials. Dig Endosc 34:451–458. 10.1111/den.14140 34536972 10.1111/den.14140

[26] Osland E, Yunus RM, Khan S, Memon MA (2011) Early versus traditional postoperative feeding in patients undergoing resectional gastrointestinal surgery: a meta-analysis. JPEN J Parenter Enteral Nutr 35:473–487. 10.1177/0148607110385698 21628607 10.1177/0148607110385698

[27] Farach SM, Danielson PD, McClenathan DT, Wilsey MJ, Chandler NM (2015) Endoscopic closure of persistent gastrocutaneous fistula in children. Pediatr Surg Int 31:277–281. 10.1007/s00383-014-3646-z 25479709 10.1007/s00383-014-3646-z

[28] Schluckebier D, Afzal NA, Thomson M (2022) Therapeutic upper gastrointestinal endoscopy in pediatric gastroenterology. Front Pediatr 9:715912. 10.3389/fped.2021.715912 35280448 10.3389/fped.2021.715912 PMC8913901

[29] McCann ME, Soriano SG (2019) Does general anesthesia affect neurodevelopment in infants and children? BMJ 367:l6459. 10.1136/bmj.l6459 31818811 10.1136/bmj.l6459

[30] O’Leary JD, Warner DO (2017) What do recent human studies tell us about the association between anaesthesia in young children and neurodevelopmental outcomes? Br J Anaesth 119:458–464. 10.1093/bja/aex141 28969310 10.1093/bja/aex141

[31] Davidson AJ, Disma N, de Graaff JC, Withington DE, Dorris L, Bell G, Stargatt R, Bellinger DC, Schuster T, Arnup SJ, Hardy P, Hunt RW, Takagi MJ, Giribaldi G, Hartmann PL, Salvo I, Morton NS, von Ungern Sternberg BS, Locatelli BG, Wilton N, Lynn A, Thomas JJ, Polaner D, Bagshaw O, Szmuk P, Absalom AR, Frawley G, Berde C, Ormond GD, Marmor J, McCann ME, GAS consortium (2016) Neurodevelopmental outcome at 2 years of age after general anaesthesia and awake-regional anaesthesia in infancy (GAS): an international multicentre, randomised controlled trial. Lancet 387:239–250. 10.1016/S0140-6736(15)00608-X 26507180 10.1016/S0140-6736(15)00608-X PMC5023520

[32] Hartjes KT, Dafonte TM, Lee AF, Lightdale JR (2021) Variation in pediatric anesthesiologist sedation practices for pediatric gastrointestinal endoscopy. Front Pediatr 9:709433. 10.3389/fped.2021.709433 34458212 10.3389/fped.2021.709433 PMC8385768

[33] Coté CJ, Wilson S, American Academy of Pediatrics, American Academy of Pediatric Dentistry (2019) Guidelines for monitoring and management of pediatric patients before, during, and after sedation for diagnostic and therapeutic procedures. Pediatrics 143:e20191000. 10.1542/peds.2019-1000 31138666 10.1542/peds.2019-1000

[34] Lee MC (2014) Sedation for pediatric endoscopy. Pediatr Gastroenterol Hepatol Nutr 17:6–12. 10.5223/pghn.2014.17.1.6 24749082 10.5223/pghn.2014.17.1.6 PMC3990786

[35] Donatelli G, Cereatti F, Dhumane P, Vergeau BM, Tuszynski T, Marie C, Dumont J-L, Meduri B (2016) Closure of gastrointestinal defects with Ovesco clip: long-term results and clinical implications. Therap Adv Gastroenterol 9:713–721. 10.1177/1756283X16652325 27582884 10.1177/1756283X16652325 PMC4984331

[36] Ovesco OTSCⓇClip—Synectics Medical Ltd. https://synecticsmedical.co.uk/product/ovesco-otsc-clip/. Accessed 13 Jun 2024

